# A Unique Case of Invasive Adenocarcinoma of the Body and Tail of the Pancreas Presenting as Gastric Outlet Obstruction

**DOI:** 10.7759/cureus.23154

**Published:** 2022-03-14

**Authors:** Syed Salman Hamid Hashmi, Rhonda-Kaye Trusty, Maria C Fonseca Mora, Ashraf Abushahin, Harry Winters

**Affiliations:** 1 Internal Medicine, NYU Langone, Woodhull Medical Center, New York, USA; 2 Gastroenterology, New York Medical College, Metropolitan Hospital Center, New York, USA; 3 Gastroenterology, NYU Langone, Woodhull Medical Center, New York, USA

**Keywords:** acquired duodenal stricture, ischemic gastritis, gastric body necrosis, adenocarcinoma of pancreas, malignant gastric outlet obstruction

## Abstract

Advanced-stage pancreatic cancer can present as secondary gastric outlet obstruction (GOO), which is an extremely rare entity. Given the initial vague presentation of gastrointestinal symptoms, the diagnosis is often delayed, leading to high morbidity and mortality. We report the case of a 68-year-old male patient who presented with vomiting, epigastric pain, and weight loss. CT abdomen and pelvis showed a distended stomach with a transition point in the duodenum. Immediate stomach decompression through the nasogastric tube was performed. Upper endoscopy (EGD) revealed ischemic gastritis with gastric body necrosis. Biopsy of the duodenum revealed moderately differentiated invasive adenocarcinoma. Magnetic resonance cholangiopancreatography (MRCP) showed a 7-cm mass centered in the body and the tail of the pancreas, invading the duodenojejunal junction. No surgical or oncological management was indicated due to the advanced stage of the malignancy at the time of the diagnosis. Malignant GOO, even though rare, should be a part of the differential diagnosis in elderly patients with vague gastrointestinal symptoms.

## Introduction

Gastric outlet obstruction (GOO) in elderly patients is concerning for malignancy. GOO, especially in advanced stages, can be life-threatening as it might cause malnutrition, electrolyte imbalance, and dehydration [1,2]. Although extremely rare, advanced-stage pancreatic cancer can lead to secondary GOO, mainly by metastasis. Often, the diagnosis is missed or delayed due to vague symptomatology, which increases the rate of morbidity and mortality. Patients in the late stages of malignant GOO have a poor prognosis. Most of the patients have a low quality of life with recurrent nausea and vomiting. Palliative care is considered the mainstay of treatment. The primary goal is to improve the quality of life of the patient by providing nourishment and controlling nausea and vomiting [2]. We report a unique case of pancreatic adenocarcinoma that presented as GOO.

## Case presentation

**Figure 1:**
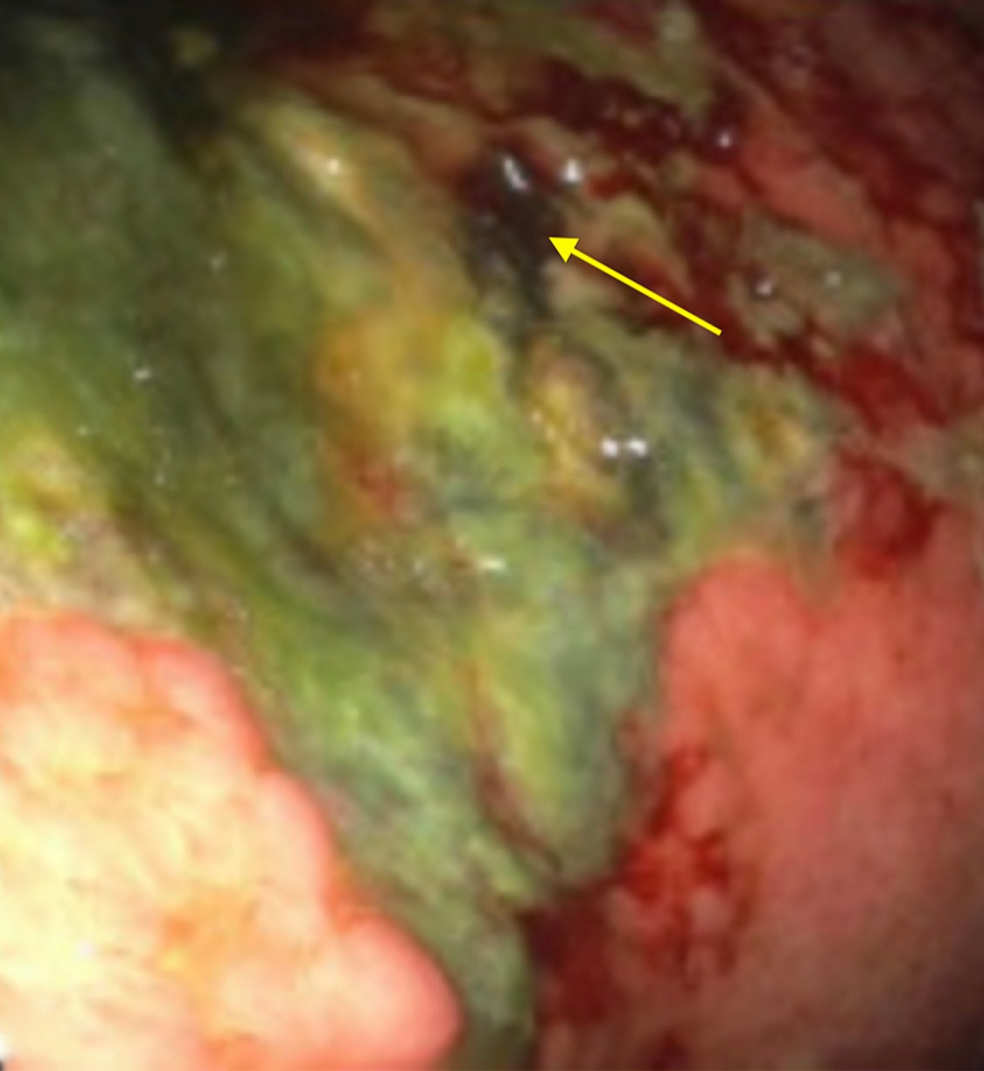
The image of gastroduodenoscopy; the yellow arrow shows the area of gastric body necrosis

**Figure 2:**
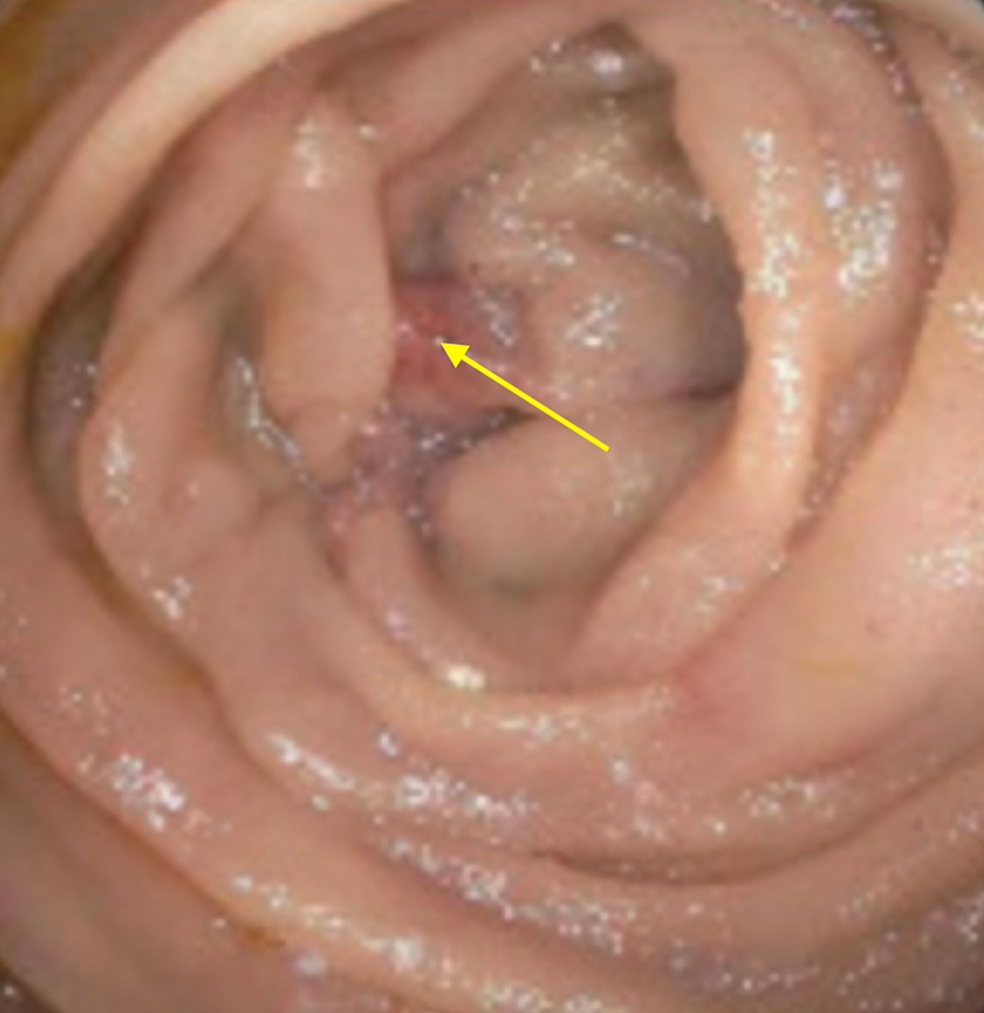
The image of gastroduodenoscopy that shows duodenal stricture; the yellow arrow shows the distal area of ischemia

A 68-year-old male presented with epigastric pain and non-bloody, non-bilious vomiting for three days. He endorsed loss of appetite, 20 pounds of unintentional weight loss, constipation, and weakness in the last two months. On presentation, the patient was noted to be in atrial fibrillation complicated by hypotension. On exam, the patient was lethargic with cold and clammy extremities; no abdominal tenderness or palpable masses were appreciated. His labs were significant for elevated lactate (10.4 mmol/L, reference range: >2 mmol/L) and a new cholestatic pattern of liver injury [aspartate aminotransferase (AST): 113 U/L, reference range: 10-40 U/L; alanine aminotransferase (ALT): 209 U/L, reference range: 7-56 U/L; total bilirubin: 14.5 mg/dL, reference range: 0.1-1.2 mg/dL]. CT abdomen and pelvis showed a distended stomach with a transition point in the duodenum. Immediate stomach decompression through the nasogastric tube was started, and the patient was transferred to the ICU for septic shock secondary to suspected bowel obstruction. He was started on cefepime and vancomycin. Upper endoscopy (EGD) revealed ischemic gastritis with gastric body necrosis (Figure 1), severe esophagitis, and an acquired duodenal stricture (Figure 2).

**Figure 3:**
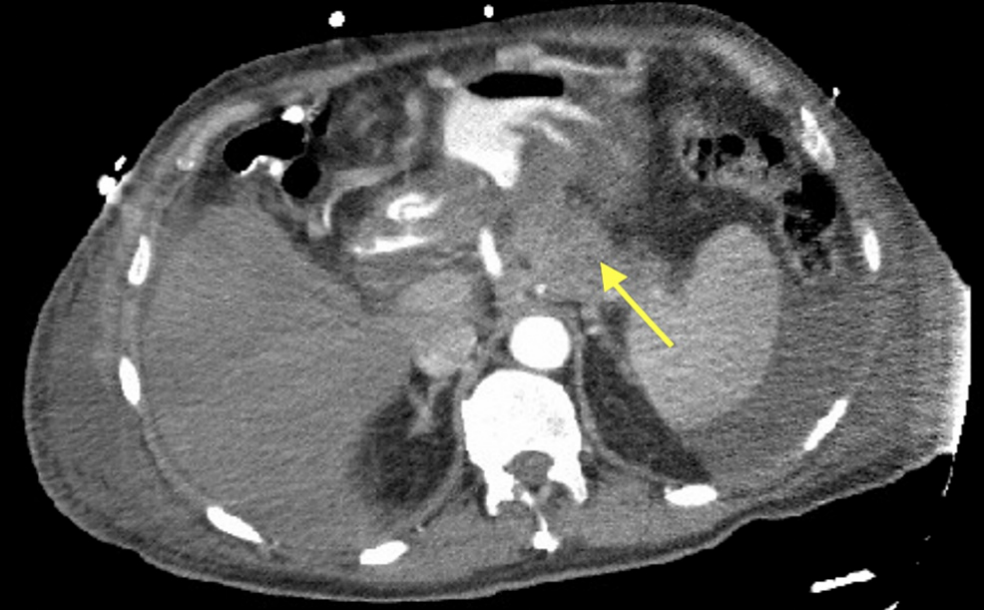
CT angiography of the abdomen and pelvis with contrast; the yellow arrow shows a hypodense mass in the body and the tail of the pancreas CT: computed tomography

CT angiogram of the abdomen and pelvis showed a hypodense mass in the body and the tail of the pancreas (Figure 3), but there were no findings of mesenteric ischemia. Biopsy of the duodenum revealed moderately differentiated invasive adenocarcinoma. Magnetic resonance cholangiopancreatography (MRCP) showed a 7-cm mass centered in the body/tail, invading the duodenojejunal junction and inferior stomach, with occlusion of the portal splenic confluence; long-segment proximal celiac axis encasement was noted. CT chest revealed pulmonary nodules suggestive of further metastasis. After extensive discussion with surgical oncology, gastroenterology, and radiation oncology, the patient was deemed ineligible for surgery given the vascular invasion and metastasis. He was also not considered for chemotherapy or radiotherapy due to severe hyperbilirubinemia. Additionally, stenting was not an option due to the absence of significant obstruction. Palliative care was consulted and the patient was discharged to hospice care.

## Discussion

Of note, 15-20% of pancreatic cancer patients develop GOO [3]. Tumors of the body and the tail of the pancreas account for 33% of the pancreatic neoplasm, and they are associated with a poor prognosis due to their late presentation [4,5]. Our case report highlighted a rare presentation of pancreatic neoplasm of the body and the tail that caused GOO, which is seen more commonly in the neoplasm of the head of the pancreas. Advanced obstruction generally presents with non-bilious vomiting and nausea and is caused by the direct compression of the tumor on the duodenum [1,2]. The proximal and distal duodenum are very susceptible to compression from the masses in the pancreas.

Patients with malignant GOO have a low life expectancy, and a multidisciplinary approach that includes palliative care, surgery, and gastroenterological and oncological management is needed to form a care plan. Traditionally, GOO was treated surgically with an open gastrojejunostomy (GJ), but laparoscopic GJ has been shown to have fewer complications [4]. Endoscopic modalities like duodenal stents are associated with faster relief of symptoms [3]. The palliative endoscopic stent is very well tolerated by GOO patients and was first used in the 1990s. Palliative stents have very low rates of complications, which include perforation, aspiration pneumonia, and hemorrhage [3]. GOO management includes pain control, decompression, and providing adequate nutrition via stent placement or surgical intervention [4,5]. Determining if surgical intervention is warranted is based on the patient's burden of disease and prognosis. Though radiation therapy has a delayed response, it is considered a treatment option for malignant obstruction. Stenting or endoscopic placement of a feeding tube can provide nourishment until a response from radiation therapy is achieved.

## Conclusions

GOO may prove to be life-threatening as it causes severe disruption in vasculature, solid organs, and viscera, making patients prone to hypotension, multifactorial shock, and numerous metabolic derangements. Although malignant GOO is rare, it should be part of the differential diagnosis in elderly patients with vague gastrointestinal symptoms, and a lower threshold for abdominal imaging may be beneficial in such patients.
